# Effect of diclofenac etalhyaluronate (SI-613) on the production of high molecular weight sodium hyaluronate in human synoviocytes

**DOI:** 10.1186/s12891-019-2586-0

**Published:** 2019-05-10

**Authors:** Tomochika Kisukeda, Junichi Onaya, Keiji Yoshioka

**Affiliations:** Central Research Laboratories, Research & Development Division, Seikagaku Corporation, 1253, Tateno 3-chome, Higashiyamato-shi, Tokyo 207-0021 Japan

**Keywords:** Osteoarthritis, Hyaluronan, Diclofenac etalhyaluronate, Hyaluronan synthase, Hyaluronidase

## Abstract

**Background:**

We have reported that a single intra-articular injection of diclofenac etalhyaluronate (SI-613) exerted a potent and long-lasting analgesic effect in experimental arthritis models. In the present study, we investigated the effect of SI-613 on the production of high molecular weight hyaluronic acid (HMW-HA) in synoviocytes from osteoarthritis (OA) patients and compared its efficacy with that of hyaluronic acid (HA).

**Methods:**

We compared the effect of SI-613, HA, and diclofenac sodium (DF-Na) on high molecular weight HA production by human synoviocytes.

**Results:**

SI-613 and exogenous HA induced the production of high molecular weight HA in synoviocytes from OA patients, whereas DF-Na had no effect. The molecular weight of newly produced HA was about 1000 kDa in the HA-treated synoviocytes and much higher than 2400 kDa in the SI-613-treated cells. The effect of the mixture of HA and DF-Na was similar to that of HA alone in that the molecular weight of newly produced HA was around 1000 kDa. SI-613 significantly suppressed hyaluronidase 2 (HYAL2) mRNA expression and significantly enhanced hyaluronan synthase 2 (HAS2) mRNA expression. HA had no effect on the expression levels of HYAL and HAS.

**Conclusion:**

The present results clearly demonstrate that SI-613 induces the production of high molecular weight HA in synoviocytes from OA patients, suggesting the long-lasting analgesic and disease modifying effect of SI-613 for OA. Taken together with the anti-inflammatory and analgesic effects we recently reported for the intra-articular administration of SI-613 to experimental animal models, SI-613 holds great promise for the treatment of knee osteoarthritis.

## Background

Osteoarthritis (OA) is the most common joint disorder worldwide and one of the leading causes of disability in the elderly [1]. Treatment for knee OA aims to relieve pain and improve function in order to mitigate reductions in physical activity. Intra-articular (IA) injection of hyaluronic acid (hyaluronan, HA), which is a linear glycosaminoglycan composed of repeating disaccharides of glucuronic acid and *N*-acetylglucosamine, is a recognized treatment for pain associated with symptomatic knee OA [2–4]. HA is distributed throughout the body, and especially in synovial fluid [5, 6]. Balazs reported that the molecular weight of HA in the synovial fluid of OA patients was much lower than that of healthy humans [6]. Dahl et al. reported that the HA concentration in synovial fluid and its molecular weight in patients with OA and rheumatoid arthritis (RA) are lower than those in healthy subjects [7]. In the knee joint, HA plays a major role in maintaining the lubricant properties of synovial fluid under both dynamic and static conditions, and in exerting various physiological activities such as reduction of pro-inflammatory cytokine levels for mitigating cartilage degeneration, and reduction of COX-2 production for reducing pain sensation [8–10]. Therefore, it was anticipated that intra-articular injection of HA would be beneficial for the management of knee OA. Notably, Asari et al. reported that positive staining of HA was distinctly reduced in synovial lining cells following anterior cruciate ligament (ACL) transection in dogs, but HA staining was maintained in the synovial lining cells of the HA-treated animals [11]. Furthermore, Ikeya et al. reported that exogenous HA induced the production of endogenous HA in synoviocytes from OA and RA patients [12]. The increase in endogenous HA is considered to normalize pathological synovial fluid and contributes to a long-term analgesic effect.

In pursuit of next generation OA therapeutics, we developed a novel conjugated compound, SI-613, which is a derivative of high-molecular-weight HA produced by fermentation (600,000 to 1200,000 Da) tethered to the non-steroidal anti-inflammatory drug diclofenac (DF) via a 2-aminoethanol linker extended from the glucuronic acid moieties [13]. SI-613 releases DF locally in a sustained manner and remains in the joint for a long period, similar to the existing IA-HA injection formulation. We previously reported that intra-articularly administered SI-613 exerts much more robust anti-inflammatory and analgesic effects than native HA or orally-administered DF-Na in experimental animal models [13]. SI-613 is thus a promising candidate in clinical development for symptomatic knee OA.

In the present study, we investigated the effects of SI-613 on the production of high molecular weight HA in synoviocytes from OA and RA patients and compared these effects with those of HA. Furthermore, we clarified the mechanism by which SI-613 induces the production of high molecular weight HA.

## Methods

### Materials

ARTZ Dispo® (1% HA) and hyaluronidase were purchased from Seikagaku Corporation (Tokyo, Japan). Diclofenac sodium was from Wako Pure Chemical Industries, Ltd. (Osaka, Japan). Water for injection (WFI) was from Otsuka Pharmaceutical Factory, Inc. (Tokushima, Japan). Human fibroblast-like synoviocytes from rheumatoid arthritis (HFLS-RA) patients and those from osteoarthritis (HFLS-OA) patients were purchased from Cell Applications, Inc. (San Diego, CA). Growth medium and a subculture reagent kit were from Cell Applications, Inc. α-MEM, α-MEM powder, penicillin/streptomycin and Dulbecco’s phosphate buffered saline (D-PBS) were from Life Technologies Corporation (Waltham, MA). FBS was from MP Biomedicals LLC. (Santa Ana, CA). IL-1β was from R&D Systems, Inc. (Minneapolis, MN). Bovine serum albumin (BSA) was from Sigma-Aldrich Co. LLC. (St. Louis, MO). Glucosamine hydrochloride, D-[6-3H (N)] ([3H]glucosamine) (37 MBq/mL) and Ultima-FloTMM (scintillation solution) were from PerkinElmer Co., Ltd. (Waltham, MA). OH pak SB-805 HQ and OH pak SB-807 HQ columns were from SHOWA DENKO K.K. (Tokyo, Japan). Select-HATM 500 k, Select-HATM 1000 k, and Select-HATM 2500 k were from Hyalose, LLC. (Oklahoma City, OK).

### Preparation of SI-613

SI-613 was prepared by conjugating high-molecular-weight fermented HA (600,000 to 1200,000 Da) and DF via a 2-aminoethanol linker extended from the glucuronic acid moieties. The SI-613 active pharmaceutical ingredient was manufactured in accordance with good manufacturing practice (GMP) guidelines, and its solution was prepared in a laminar flow cabinet to maintain its sterility. SI-613 was dissolved in 5 mM phosphate-buffered saline (pH 6.0) at a concentration of 10 mg/mL and diluted appropriately with PBS before use. Although SI-613 has been used at 10 mg/mL in clinical trials, the maximum concentration of SI-613 was set at 1 mg/mL in this study because highly-concentrated SI-613 solution is very viscous and difficult to pipette.

### Cells and cell culture

Three lots of human fibroblast-like synoviocytes from rheumatoid arthritis (HFLS-RA) patients and three from osteoarthritis (HFLS-OA) (Cell Applications, Inc.) patients were cultured separately in basal medium containing 10% growth supplement and 1% penicillin/streptomycin (Cell Applications, Inc.). Cells were incubated at 37 °C under 5% CO_2_. The medium was changed every 2 days. After confluence, the cells were seeded at a density of 3.0 × 10^5^ cells/2 mL/well in α-MEM medium containing 10% fetal bovine serum (FBS) and 1% penicillin/streptomycin in 6-well plates.

### Challenge to test materials

One day after cell seeding, the culture medium was replaced with 2 mL of α-MEM medium containing 10% FBS, 10 ng/mL recombinant human IL-1β/IL-1F2 (IL-1β) (R&D Systems, Inc.), 1% penicillin/streptomycin, 370 kBq/mL glucosamine hydrochloride D-[6-^3^H (N)] ([^3^H]glucosamine) and each test material: HA (ARTZ Dispo®), SI-613 (Seikagaku Corporation), diclofenac sodium (DF-Na) (Wako Pure Chemical Industries, Ltd.), or a mixture of DF-Na and HA (DF-Na + HA). For mRNA expression measurements, [^3^H] glucosamine was excluded from the medium. The cells were incubated at 37 °C for the indicated periods under 5% CO_2_.

### Collection of the culture supernatant and cell lysate

Culture supernatant fraction for the measurement of high molecular weight HA (HMW-HA, MW > 2400 kDa) production was collected at each incubation time. Cell lysate for the measurement of RNA was collected at 48 h and stored frozen at -80 °C.

### Fractionation of the culture supernatant and measurement of the radioactivity levels

Radiolabeled HA in the culture supernatant was separated using a size exclusion HPLC column (an OH pak SB-805 HQ column or an OH pak SB-807 HQ column) in an HPLC system (JASCO Corporation). HPLC was performed at 0.5 mL/min of mobile phase (5 mmol/L phosphate buffer, 0.82% NaCl: acetonitrile = 2: 1) at 35 °C. The injection volume of culture supernatant was set at 10 μL. Eluent was collected continuously as 0.5 min fractions from 9 min (OH pak SB-805 HQ column) or 14 min (OH pak SB-807 HQ column) after starting the elution using a fraction collector (JASCO Corporation). Liquid scintillator (Ultima-FloTMM, PerkinElmer Co., Ltd.) was added to each HPLC fraction and mixed well. The disintegrations per minute (dpm) of each fraction were measured in a liquid scintillation counter (PerkinElmer Co., Ltd.). Each sample was analyzed in a single HPLC run.

### Evaluation of the molecular weight of HA in each fraction using HA standard solutions

Select-HA™ 500 k, Select-HA™ 1000 k and Select-HA™ 2500 k, for which weight-average molecular weights were 528 kDa, 1076 kDa, and 2420 kDa respectively, were used for molecular weight calibration. Each standard was subjected to size exclusion HPLC analysis with online monitoring of UV absorption at 210 nm. The peak top fraction of each HA standard was calculated by considering the lag between the UV monitor and the fraction collector.

### Enzyme digestion

The culture supernatant of the 0.01% HA-treated group (90 μL) was mixed with 10 μL of 100 turbidity reducing units (TRU)/mL hyaluronidase or WFI and incubated overnight at 37 °C, except for the culture supernatant of the 0.01% SI-613-treated group, which was digested by hyaluronidase treatment (30 μL hyaluronidase solution at 60 °C for 3 h). This more aggressive treatment was required because HA in the culture supernatant of the 0.01% SI-613-treated group was more difficult to digest.

### Cell counting

Immediately after collecting the culture supernatant, each well was washed with Dulbecco’s phosphate-buffered saline (D-PBS) (GIBCO, Life Technologies Corporation). Trypsin/EDTA solution (Cell Applications, Inc.) was added to the wells and the cells were incubated at 37 °C under 5% CO_2_. The cells were detached from the wells by tapping the plate, and 0.5 mL of trypsin-neutralizing solution (Cell Applications, Inc.) was added. The live cells were counted using a hematocytometer after staining with trypan blue.

### RNA extraction and cDNA sample preparation

RNA was extracted from the cell lysates according to the instruction manual for the RNeasy plus mini kit (QIAGEN). RNA concentrations were measured by ultramicro spectrophotometry (Thermo Fisher Scientific, Inc.). The RNA was stored frozen at -80 °C. cDNA samples were prepared using the Super Script III First-Strand Synthesis System (Invitrogen, Life Technologies Corporation).

### Real-time quantitative PCR

The mRNA expression levels of the target genes (HAS1, HAS2, HAS3, HYAL1, HYAL2, and HYAL3) and GAPDH were measured in duplicate using real-time quantitative polymerase chain reaction (PCR; RT-qPCR). RT-qPCR was performed using TaqMan Gene Expression Assay (TaqMan) (Applied Biosystems) and Premix Ex Taq (Perfect Real Time, Takara Pac LTD.) according to the instruction manuals. The following sets of TaqMan probes and primers were used to measure the expression levels of the target genes: HAS1 (ID: Hs00987418_m1), HAS2 (ID: Hs00193435_m1), HAS3 (ID: Hs00193436_m1), HYAL1 (ID: Hs00201046_m1), HYAL2 (ID: Hs01117343_g1), HYAL3 (ID: Hs00185910_m1), and GAPDH (ID: Hs03929097_g1). Reagents were aliquoted into each well at the following volumes: 1 μL of TaqMan, 12.5 μL of Premix Ex Taq, 2 μL of ROX II (× 40), and 7.5 μL distilled water. Next, 2 μL of the cDNA sample was added. The PCR conditions were as follows: first denaturation at 95 °C for 30 s., followed by a 40-cycle sequence of denaturation (95 °C, 5 s.), annealing (60 °C, 30 s.), and extension (72 °C, 20 s.). The cycle threshold (Ct) value was measured for each gene using a real-time PCR system (MX3000P; Stratagene Corporation). The Ct value was analyzed using the comparative Ct method (^ΔΔ^Ct method) to calculate the target mRNA expression level, followed by normalization to GAPDH level. The relative mRNA expression levels were presented as the times-fold increase compared to that of the control.

## Statistical analyses

Statistical analyses were performed using the Statistical Analysis System, SAS (SAS Institute Inc., Cary, NC). Radioactivity levels divided into 2 groups with different molecular weight ranges were analyzed by parametric Dunnett’s test. The GAPDH-normalized mRNA expression levels in the control, HA, and SI-613 groups were analyzed by using parametric Dunnett’s test for HAS and HYAL. The statistically significant level was set at 5% (two-tailed). Data were represented as the mean and standard error of the mean (SE).

## Results

### Concentration-dependent effects of HA and SI-613 on the incorporation of [^3^H] glucosamine into high molecular weight products in HFLS-RA cells

**Fig. 1:**
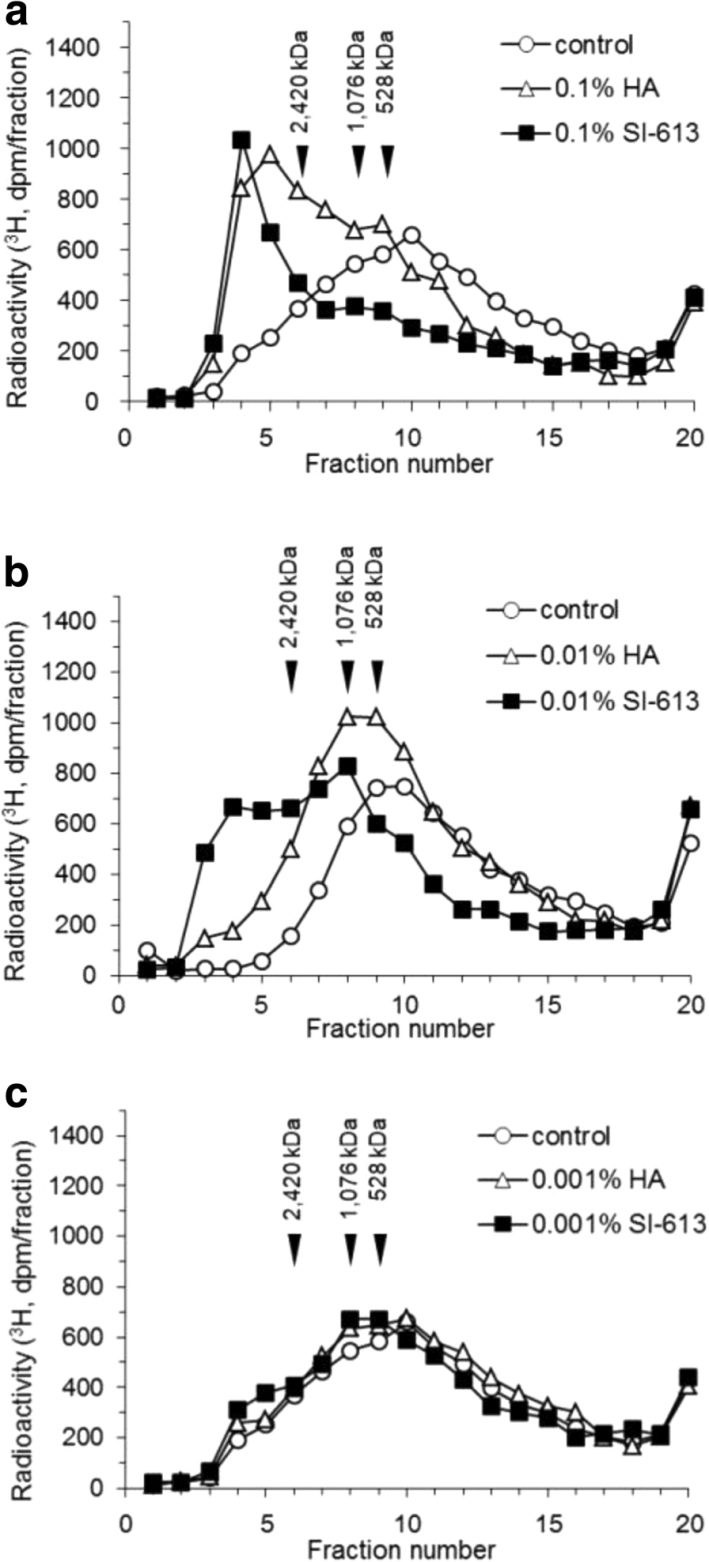
Concentration-dependent effects of HA and SI-613 on the incorporation of [^3^H] glucosamine into high molecular weight products. HFLS-RA cells were cultured in [^3^H] glucosamine and IL-1β in the absence (solid squares) or presence of HA (open triangles) and SI-613 (open circles) for 48 h. The concentrations of HA and SI-613 were set at 0.1% (**a**), 0.01% (**b**) or 0.001% (**c**). Radiolabeled HA in the culture supernatant was separated by size exclusion HPLC using an OH pak SB-805 HQ column. The data represent results from a single HPLC run with a single well

HFLS-RA cells derived from one donor were incubated with [^3^H] glucosamine and IL-1β for 48 h in the presence of HA or SI-613. Each resulting culture supernatant was then subjected to size exclusion HPLC analysis (OH pak SB-805 HQ column) followed by liquid scintillation counting. The column was calibrated using MW standards of HA. In control experiments, [^3^H] glucosamine alone was eluted at fractions later than no. 20. The culture supernatant of untreated HFLS-RA cells provided a broad peak with a peak top at fraction no. 10 (MW = 500 kDa) in addition to the [^3^H] glucosamine peak (Fig. 1a). In contrast, the culture supernatant of 0.1% HA-treated HFLS-RA cells provided a sharp peak with a peak top at fraction no. 5 (MW > 2400 kDa) and a shoulder at fraction no. 9 (MW = 1000 kDa) (Fig. 1a). The culture supernatant of SI-613-treated cells showed a much sharper peak with a peak top at fraction no. 4 (MW > 2400 kDa) (Fig. 1a).

By decreasing the dose to 0.01%, the first peak (MW > 2400 kDa) of HA-treated culture supernatant disappeared, whereas that of SI-613-treated culture supernatant was only slightly blunted (Fig. 1b). At a dose of 0.001%, the culture supernatant of treated and non-treated cells provided very similar broad peaks (Fig. 1c). These results clearly indicate that HA and SI-613 stimulated the incorporation of [^3^H] glucosamine into high molecular weight products in a dose-dependent manner, and SI-613 was much more effective in this activity than HA.

In addition, the synthesis of high molecular weight HA by synovial cells was not marked without IL-1β stimulation (data not shown). This result is presumably relevant to the findings of another study [14] that chondroitin sulfate stimulates the synthesis of HMW-HA in fibroblast-like synoviocytes following IL-1β stimulation. The underlying mechanisms remain to be elucidated.

### Hyaluronidase treatment of the culture supernatant of HFLS-RA cells

**Fig. 2:**
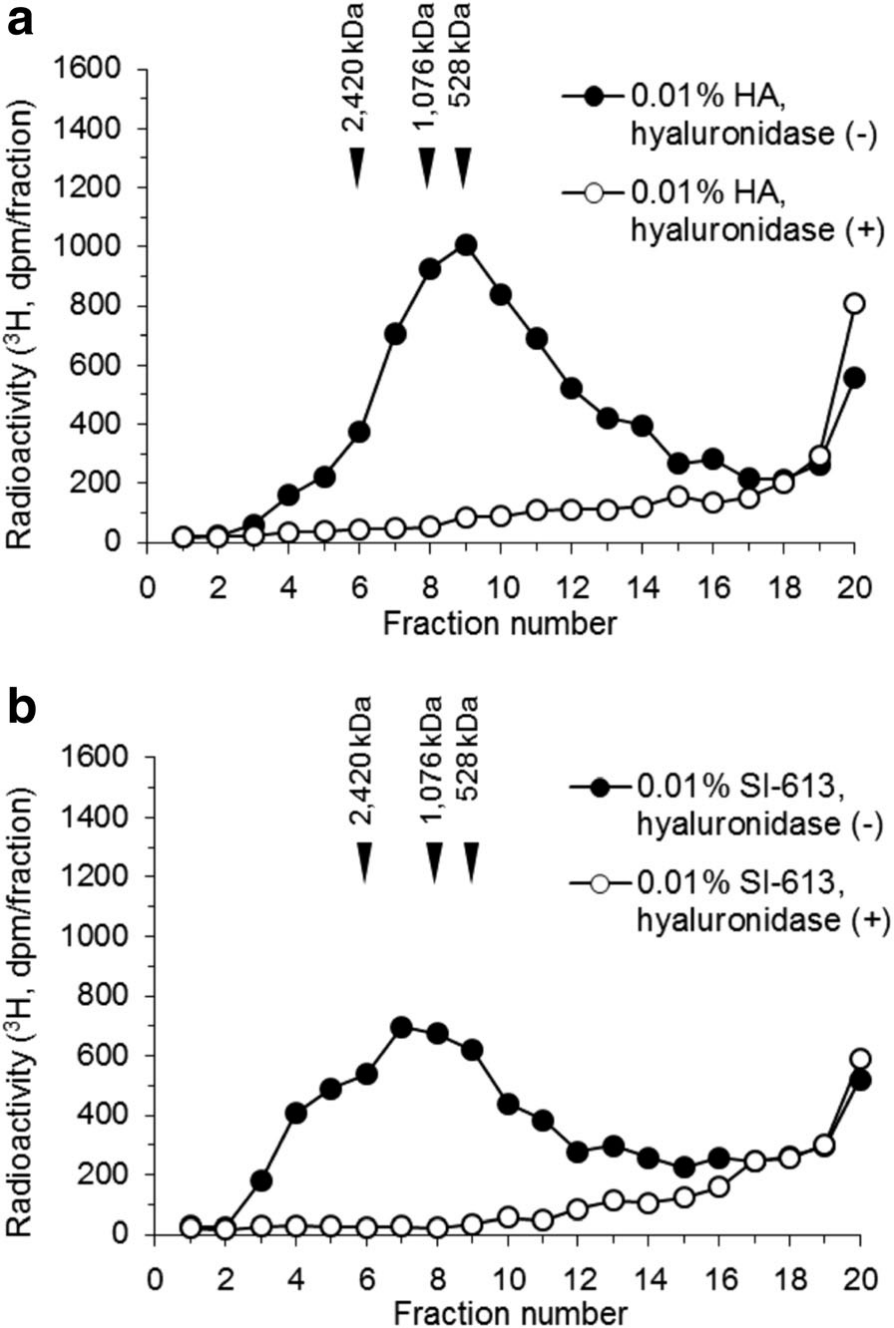
Hyaluronidase treatment of the culture supernatant of HFLS-RA cells. Culture supernatant fractions of 0.01% HA-stimulated (**a**) and 0.01% SI-613-stimulated (**b**) cells were incubated in the absence or presence of hyaluronidase, then analyzed by size exclusion HPLC using an OH pak SB-805 HQ column. The data represent results from a single HPLC run with a single well

**Fig. 3:**
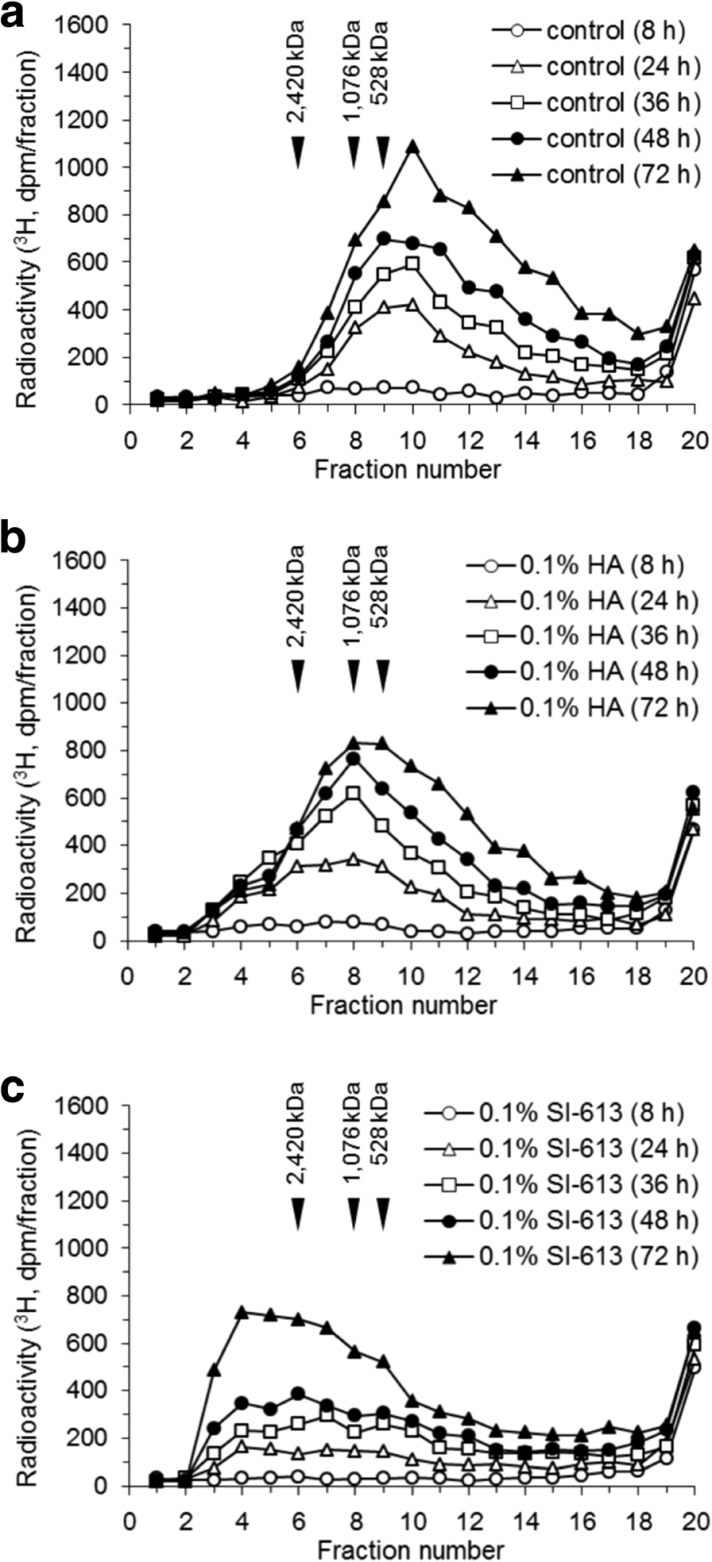
Time course of [^3^H] glucosamine incorporation into high molecular weight products. The HFLS-RA cells were cultured in [^3^H] glucosamine and IL-1β in the absence (**a**) or presence of 0.1% HA (**b**) and 0.1% SI-613 (**c**) for 8, 24, 36, 48 or 72 h. Radiolabeled HA in the culture supernatant was separated by size exclusion HPLC using an OH pak SB-805 HQ column. The data represent results from a single HPLC run with a single well

HFLS-RA cells derived from a single donor were used. To characterize the [^3^H]glucosamine-incorporated products in the culture supernatant of HA- and SI-613-treated HFLS-RA cells, the culture supernatant fractions were analyzed by gel filtration HPLC after incubation with or without hyaluronidase. As shown in Fig. 2, each peak in HA- and SI-613-treated culture supernatant disappeared upon treatment with hyaluronidase. This result demonstrates that most of the [^3^H]glucosamine-incorporated product in HFLS cells was newly synthesized HMW-HA (Fig. 3).

### Effects of SI-613 and its chemical composition on the molecular weight distribution of newly produced HA

**Fig. 4:**
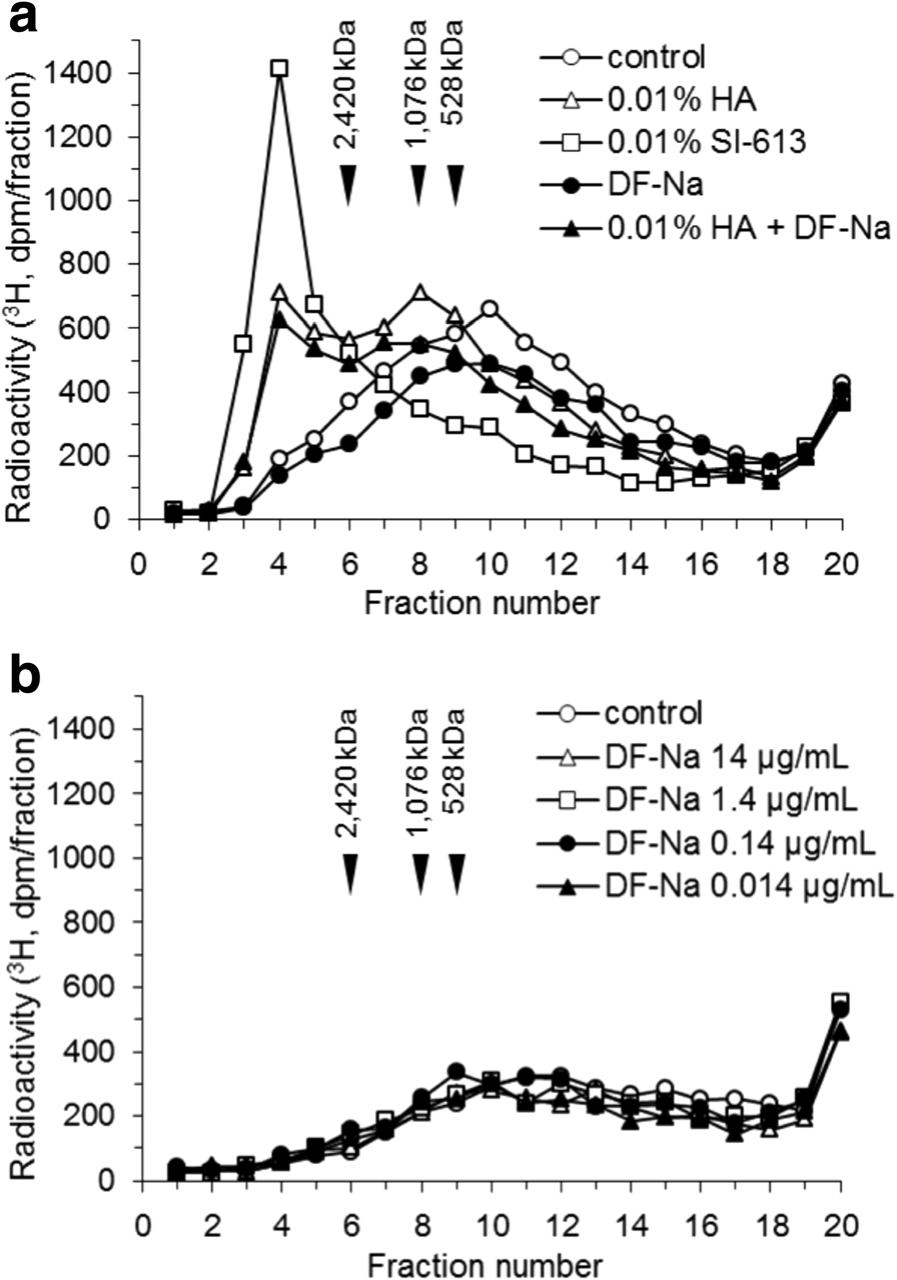
Effects of SI-613 and its chemical components on the molecular weight distribution of newly produced HA. HFLS-RA cells were cultured in [^3^H] glucosamine and IL-1β with the test materials for 48 h. Radiolabeled HA in the culture supernatant was separated by size exclusion HPLC using an OH pak SB-805 HQ column. **a**, The test materials were PBS (control), 0.01% HA, 0.01% SI-613, 1.4 μg/mL DF-Na or a mixture of 1.4 μg/mL DF-Na and 0.01% HA (DF-Na + HA). **b**, The test materials were 14, 1.4, 0.14 or 0.014 μg/mL DF-Na. The data represent results from a single HPLC run with a single well

HFLS-RA cells derived from a single donor were used. The addition of SI-613 to HFLS-RA cells resulted in the conversion of most newly produced HA to HMW-HA of > 2400 kDa. The distribution of molecular weights of newly produced HA was different following SI-613- and HA-treatment. To clarify the relationship between SI-613 and the production of HMW-HA, the effect of SI-613 was compared with that of each chemical component of SI-613: HA, DF-Na, and a mixture of DF-Na and HA (DF-Na + HA). For DF-Na, we tested four concentrations (0.014–14 μg/mL), which corresponded to 0.1–100% liberation of DF-Na from a 0.01% SI-613 solution. This study was necessary due to the lack of accurate data for liberated DF-Na in cell cultures, although it was known that 10% of the DF-Na in SI-613 was liberated by incubation at 37 °C for 48 h in cell-free medium (data not shown). We found that the HMW-HA produced by stimulation with HA was similar to that induced by DF-Na + HA (Fig. 4a). Most of the radioactivity incorporated in the presence of DF-Na was found in later-eluted fractions containing low molecular weight compounds of less than 500 kDa, similar to the control (Fig. 4a, b). HMW-HA produced by stimulation with SI-613 was eluted as a sharp peak, which was a completely different pattern from that obtained with the other conditions (Fig. 4a). This result indicates that the anabolic effect of SI-613 in producing HMW-HA was qualitatively different from that produced by its chemical components.

### SI-613 produced an increase in high molecular weight HA in human fibroblast-like synoviocytes from rheumatoid and osteoarthritis patients

**Fig. 5:**
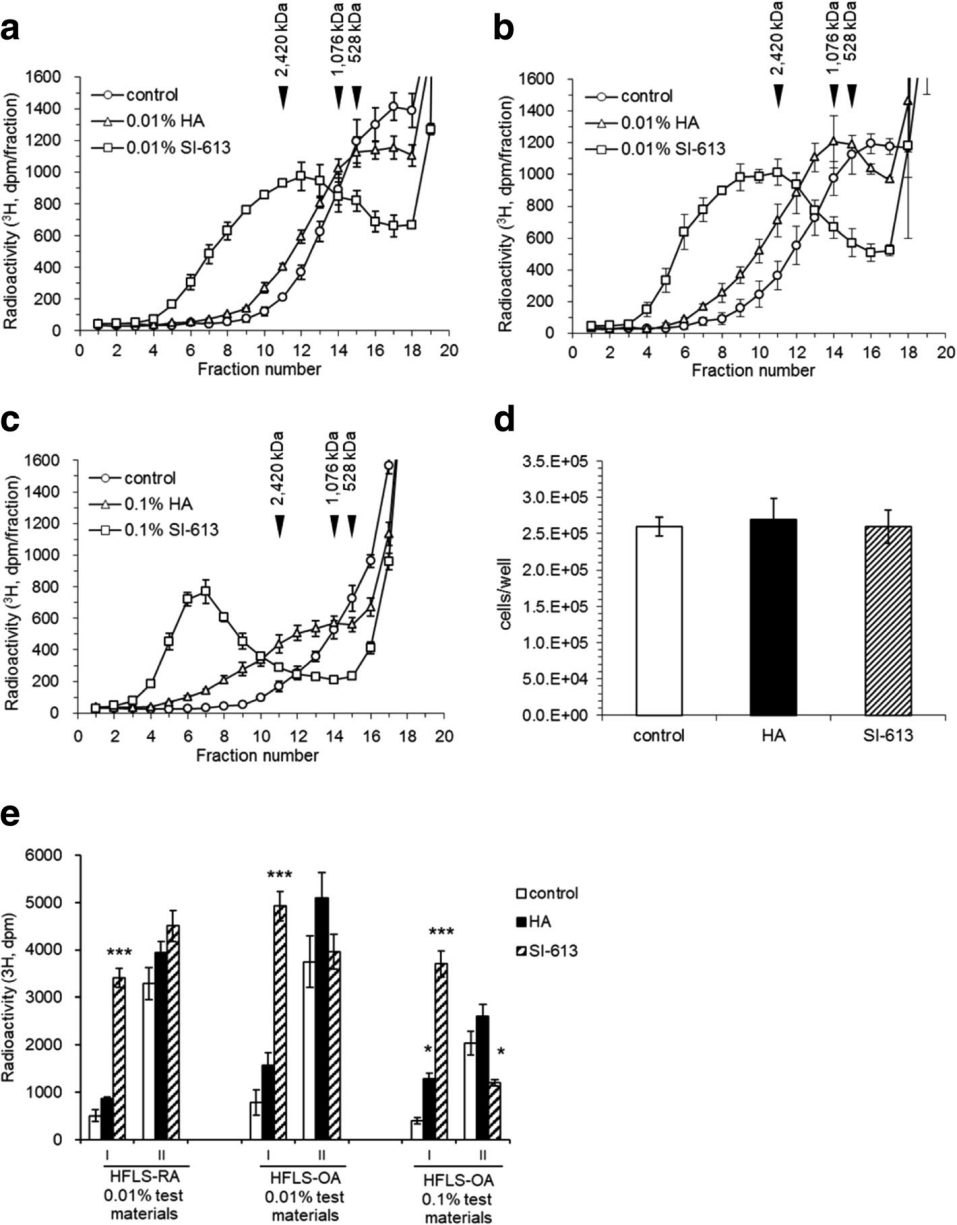
SI-613 induced increased production of high molecular weight HA in HFLS-RA and HFLS-OA cells. HFLS-RA cells (**a**) and HFLA-OA cells (**b**, **c**, **d**) from three donors were separately cultured in [3H] glucosamine and IL-1β with HA or SI-613 at 0.01% (**a**, **b**) or 0.1% (**c**, **d**) or with PBS for 48 h. Cultures from individual patients were maintained as separate cultures and cells were not pooled. Radiolabeled HA in the culture supernatant was separated using an OH pak SB-807 size exclusion HPLC column (**a**–**c**). Each value is from a single HPLC run with a single well. The number of HFLS-OA cells after collection of the culture supernatant was counted (**d**). Values are represented as the means ± S.E. of three donors. Parametric Tukey’s test was used for statistical analysis. The data from **a**, **b** and **c** were re-analyzed by dividing the fractions into 2 groups with different molecular weight ranges (range I, MW > 2400 kDa; range II, MW = 500–2400 kDa). The fractions eluted later than the 500 kDa MW marker were excluded from the re-analysis because most of the radioactivity in these fractions is derived from free [^3^H] glucosamine (**e**). Parametric Dunnett’s test was used for statistical analyses (*P < 0.05, ***P < 0.001)

In the above studies, size exclusion HPLC analyses were conducted using an OH pak SB-805 HQ column with an optimum separation range of 100–1000 kDa. Using this separation condition, we observed a slight difference in the elution positions of newly synthesized HA between the culture supernatant fractions of HA and SI-613 treated HFLS-RA cells. To more precisely discriminate the molecular weights of HA products obtained following HA and SI-613 treatment, we performed size-exclusion HPLC using an OH pak SB-807 HQ column, which is specifically designed to separate compounds in the molecular weight range of 500–500,000 kDa. The culture supernatant of 0.01% SI-613-treated cells provided a peak of HMW-HA > 2400 kDa. This peak was clearly separated from the peak obtained when cells were stimulated by 0.01% HA (Fig. 5a), whereas the peaks obtained for HA-treated and untreated cells were poorly resolved. In synovial fibroblasts derived from three OA donors (HFLS-OA cells), SI-613 at 0.01% (Fig. 5b) and 0.1% (Fig. 5c) shifted the HPLC peak of HMW-HA dose-dependently to earlier-eluted fractions, corresponding to molecular weights of over 2400 kDa. No significant difference was found in the number of cells among the control, HA, and SI-613 groups by parametric Tukey’s test (Fig. 5d), suggesting that the effect of SI-613 in enhancing the production of HMW-HA was not caused by a change in cell number. To compare the production of high molecular weight HA by statistical analyses, we categorized the radioactivity in the separated fractions (Fig. 5a, b, and c) into 2 groups with different molecular weight ranges (MW = 500–2400 kDa and MW > 2400 kDa). The results clearly show that SI-613 significantly induces the production of high molecular weight HA in synoviocytes, compared to that by HA or PBS (Fig. 5e).

### Effects of SI-613 on HAS1, HAS2, HAS3, HYAL1, HYAL2 and HYAL3 mRNA expression

**Fig. 6:**
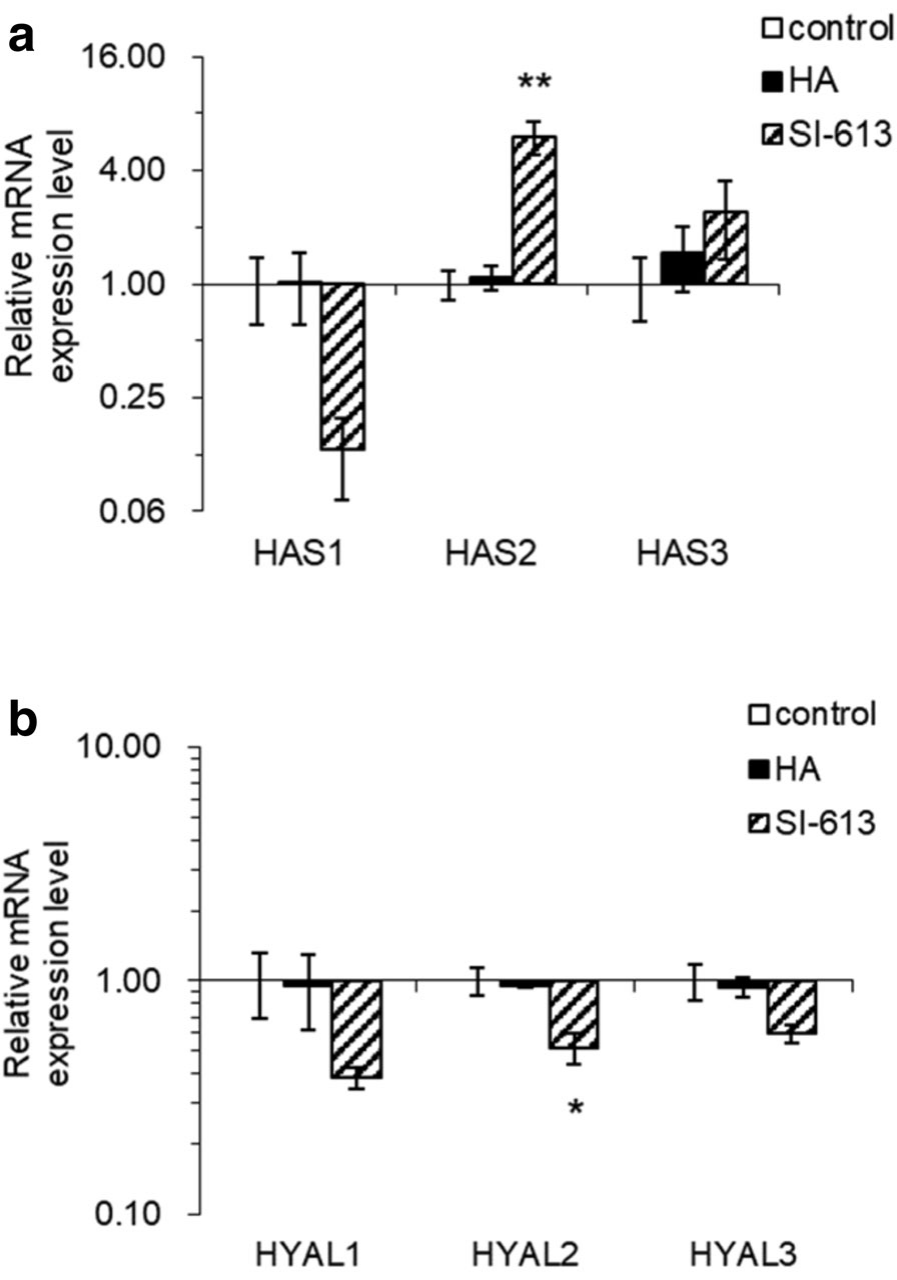
Effects of SI-613 on HAS1, HAS2, HAS3, HYAL1, HYAL2 and HYAL3 mRNA expression levels. HFLS-OA cells from three donors were cultured in IL-1β with PBS, 0.1% HA, or 0.1% SI-613 for 48 h. Cultures from individual patients were maintained as separate cultures and cells were not pooled. The mRNA expression levels of HAS1, HAS2, HAS3 (**a**), HYAL1, HYAL2 and HYAL3 (**b**) were measured by RT-qPCR. Values are represented as the means ± S.E. of three donors and shown as -fold increases in mRNA expression relative to control cells. Parametric Dunnett’s test was used for statistical analyses. *P < 0.05, **P < 0.01

HA and SI-613 were added to HFLS-OA cells from three donors in the presence of IL-1β. The mRNA expression levels of HAS1, HAS2, HAS3, HYAL1, HYAL2 and HYAL3 in the cells were evaluated using a real-time qPCR method. The mRNA expression level of HAS2 was found to be significantly increased in the SI-613 group as compared with the control group. On the other hand, the mRNA expression level of HYAL2 was significantly suppressed in the SI-613 group as compared with the control group. HA had no effect on the expression levels of HAS 1, 2, 3 and HYAL 1, 2, 3 (Fig. 6a and b).

## Discussion

We have investigated the novel HA derivative chemically linked with DF, SI-613, which is a potentially safer and more effective treatment for OA knee pain. In a previous study, we reported that a single intra-articular administration of SI-613 provided an analgesic effect via the sustained release of DF, and this pharmacological effect lasted at least 28 days [13].

In the present study, we evaluated the effect of SI-613 on the production of HMW-HA in synoviocytes from OA patients. We found that SI-613 induced the production of HMW-HA of > 2400 kDa in synoviocytes from patients with OA, whereas HA or a mixture of HA and DF-NA induced the production of HMW-HA of 1000 kDa. Although HA also produced HMW-HA of > 2400 kDa in some tests (Fig. 1a and 4a), this effect was not reproducible (Fig. 2a and 3b), perhaps because the range of column fractionation was exceeded. Using an appropriate column, we rarely detected HMW-HA > 2400 kDa in HA-treated culture supernatant (Fig. 5). DF-Na had no effect beyond basal-level HMW-HA production. We could not accurately evaluate the molecular weight of HA produced by SI-613 because there is no molecular weight standard for HA above 2420 kDa. However, the molecular weight of HA produced by SI-613 is clearly higher than 2420 kDa, and is likely comparable with the average molecular weight of HA from healthy humans, which is 6000 kDa [15]. Therefore, it appears that the stimulatory effect of SI-613 on HMW-HA production helps normalize the pathological synovial fluid of OA patients.

Our findings suggest that SI-613 exerts not only anti-inflammatory and analgesic effects due to diclofenac, but also a normalizing effect on pathological synovial fluid by producing endogenous HA. Additionally, this effect of SI-613 may contribute to a long-lasting analgesic effect.

Next, we assessed the mechanism of production of HMW-HA by SI-613. SI-613 or HA was added to the synoviocytes from OA patients to evaluate the mRNA expression levels of HA synthases (HAS1, 2 and 3) and hyaluronidases (HYAL1, 2 and 3). The results revealed that SI-613 significantly suppressed HYAL2 mRNA expression and significantly enhanced HAS2 mRNA expression. On the other hand, HA by itself did not significantly change the mRNA expression levels of HYAL1, HYAL2, HYAL3, HAS1, HAS2, and HAS3.

The abundance and molecular size of HA are thought to be controlled by HA synthases and hyaluronidases. Three isoforms (HAS1, HAS2 and HAS3) are known as human HA synthases. It has been reported that HAS1 and HAS3 are responsible for producing HA with molecular weights ranging from 200 kDa to 2000 kDa, and HAS2 synthesizes HA larger than 2000 kDa [16]. It has also been reported that HAS2 synthesizes long-chain HA larger than 3900 kDa, whereas HAS3 synthesizes various sizes of HA ranging from 120 kDa to 1000 kDa, and HAS1 synthesizes short-chain HA with a molecular weight of 120 kDa [17]. In contrast, HAYL1, 2 and 3 have been reported as human hyaluronidases. It has been reported that HYAL2 degrades HMW-HA into low-molecular (20 kDa) HA, HYAL1 degrades HA into disaccharides, and HYAL3 has no enzymatic activity and its function remains unknown [18]. Taken together with the result of this study and information reported by others, we suggest that the mechanism by which SI-613 induces production of HMW-HA larger than 2400 kDa involves the enhancement of HAS2 mRNA expression. Decreased expression of HYAL2 mRNA is also likely involved in the retention of HMW-HA by suppressing breakdown of HMW-HA. In contrast, HA did not change the expression levels of genes responsible for the synthesis and breakdown of HA, and HA and DF-Na had no effect on gene expression levels, indicating that the effect of SI-613 was specific.

The effect of exogenous HA on high molecular weight sodium hyaluronate production in synoviocytes from OA and RA patients was previously reported by Ikeya et al. However, the molecular weight of newly synthesized HA was not determined. Smith and Ghosh also reported that high molecular weight HA (MW = 4700 kDa) stimulated the HA synthesis, but low molecular weight HA (MW < 500 kDa) showed little or no effect [19]. In our study, HA with a molecular weight of 1000 kDa induced the production of only a small amount of HMW-HA (MW > 2400 kDa). In contrast, SI-613 composed of HA with a molecular weight of 600–1200 kDa induced the production of HMW-HA. This finding suggested that SI-613, i.e. DF-modified HA, probably acted in a manner similar to that by the high molecular weight HA (MW = 4700 kDa). Modification with DF may change the properties of HA (MW = 600–1200 kDa). David-Raoudi et al. have also reported that CS increases hyaluronan production in human synoviocytes through differential regulation of hyaluronan synthases via p38 and Akt. CS significantly suppressed hyaluronan synthase 3 (*HAS3*) mRNA expression and significantly enhanced *HAS2* mRNA expression. It had no effect on the expression levels of *HAS1* [14]. Our findings on the enhancement of *HAS2* mRNA expression in the present study appears to be in agreement with their findings, although the tested glycosaminoglycans are different.

In the present study, we confirmed that exogenous HA induced the production of endogenous HA with molecular weights above 1000 kDa but did not induce the production of HMW-HA > 2400 kDa or change the expression levels of HAS 1, 2, 3 and HYAL 1, 2, 3, in contrast to SI-613. Therefore, HA-induced newly synthesized HA might serve as a substitute substrate for hyaluronidase rather than as functional HA, thus preventing the digestion of high molecular weight endogenous HA.

## Conclusion

The present results clearly demonstrate that SI-613 induces the production of high molecular weight HA in synoviocytes from OA patients. In conclusion, these findings suggested that SI-613 might exert the long-lasting analgesic and disease modifying effects attributable to HA produced by synoviocytes even after the disappearance of DF. Taken together with the anti-inflammatory and analgesic effects that we recently observed following intra-articular administration of SI-613 to experimental animal models, SI-613 holds great promise for the treatment of knee osteoarthritis.

## Abbreviations

cDNA: complementary DNA
COX-2: Cyclooxygenase-2
DF: Diclofenac
HA: Hyaluronic acid
HAS: Hyaluronan synthase
HFLS: Human fibroblast-like synoviocytes
HMW: High molecular weight
HPLC: High performance liquid chromatography
HYAL: Hyaluronidase
IA: Intra-articular
mRNA: Messenger RNA
MW: Molecular weight
OA: Osteoarthritis
RA: Rheumatoid arthritis

## References

[1] Buckwalter JA, Saltzman C, Brown T (2004). The impact of osteoarthritis: implications for research. Clin Orthop Relat Res.

[2] Zhang W, Moskowitz RW, Nuki G, Abramson S, Altman RD, Arden N (2008). OARSI recommendations for the management of hip and knee osteoarthritis, part II: OARSI evidence-based, expert consensus guidelines. Osteoarthr Cartil.

[3] Zhang W, Nuki G, Moskowitz RW, Abramson S, Altman RD, Arden NK (2010). OARSI recommendations for the management of hip and knee osteoarthritis part III: changes in evidence following systematic cumulative update of research published through January 2009. Osteoarthr Cartil.

[4] Recommendations for the medical management of osteoarthritis of the hip and knee: 2000 update (2000). American College of Rheumatology Subcommittee on Osteoarthritis Guidelines. Arthritis Rheum.

[5] Balazs EA, Bloom GD, Swann DA (1966). Fine structure and glycosaminoglycan content of the surface layer of articular cartilage. Fed Proc.

[6] Balazs EA. The physical properties of synovial fluid and the special role of hyaluronic acid. In disorders of the knee. (ed. by AJ Helfet) Lippincott Co., Philadelphia, 1974, 63–75.

[7] Dahl LB, Dahl IM, Engström-Laurent A, Granath K (1985). Concentration and molecular weight of sodium hyaluronate in synovial fluid from patients with rheumatoid arthritis and other arthropathies. Ann Rheum Dis.

[8] Ogston AG, Stanier JE (1953). The physiological function of hyaluronic acid in synovial fluid; viscous, elastic and lubricant properties. J Physiol.

[9] Altman RD, Dasa V, Takeuchi J (2018). Review of the mechanism of action for Supartz FX in knee osteoarthritis. Cartilage.

[10] Santangelo KS, Johnson AL, Ruppert AS, Bertone AL (2007). Effects of hyaluronan treatment on lipopolysaccharide-challenged fibroblast-like synovial cells. Arthritis Res Ther.

[11] Asari A, Miyauchi S, Matsuzaka S, Ito T, Kominami E, Uchiyama Y (1998). Molecular weight-dependent effects of hyaluronate on the arthritic synovium. Arch Histol Cytol.

[12] Ikeya H, Miyoshi T, Nakamura T, Endo M (1994). Hyaluronate degradation and synthesis by cultured synovial fibroblasts derived from knee joints of patients suffering from rheumatoid arthritis and osteoarthritis. Connective Tissue.

[13] Yoshioka K, Kisukeda T, Zuinen R, Yasuda Y, Miyamoto K (2018). Pharmacological effects of N-[2-[[2-[2-[(2,6-dichlorophenyl)amino]phenyl]acetyl]oxy]ethyl] hyaluronamide (diclofenac etalhyaluronate, SI-613), a novel sodium hyaluronate derivative chemically linked with diclofenac. BMC Musculoskelet Disord.

[14] David-Raoudi M, Deschrevel B, Leclercq S, Galéra P, Boumediene K, Pujol JP (2009). Chondroitin sulfate increases hyaluronan production by human synoviocytes through differential regulation of hyaluronan synthases: role of p38 and Akt. Arthritis Rheum.

[15] Balazs EA, Watson D, Duff IF, Roseman S (1967). Hyaluronic acid in synovial fluid. I. Molecular parameters of hyaluronic acid in normal and arthritis human fluids. Arthritis Rheum.

[16] Itano N, Kimata K (2002). Mammalian hyaluronan synthases. IUBMB Life.

[17] Brinck J, Heldin P (1999). Expression of recombinant hyaluronan synthase (HAS) isoforms in CHO cells reduces cell migration and cell surface CD44. Exp Cell Res.

[18] Stern R (2005). Hyaluronan metabolism: a major paradox in cancer biology. Pathol Biol.

[19] Smith MM, Ghosh P (1987). The synthesis of hyaluronic acid by human synovial fibroblasts is influenced by the nature of the hyaluronate in the extracellular environment. Rheumatol Int.

